# Curcumin as a Potential Treatment for COVID-19

**DOI:** 10.3389/fphar.2021.675287

**Published:** 2021-05-07

**Authors:** Bruna A. C. Rattis, Simone G. Ramos, Mara R. N. Celes

**Affiliations:** ^1^ Department of Pathology, Faculty of Medicine of Ribeirão Preto, University of São Paulo, Ribeirão Preto, Brazil; ^2^ Department of Bioscience and Technology, Institute of Tropical Pathology and Public Health, Federal University of Goias, Goiania, Brazil

**Keywords:** curcumin, COVID-19, SARS-CoV-2, new therapies, ACE2

## Abstract

Coronavirus disease 2019 (COVID-19) is an infectious disease that rapidly spread throughout the world leading to high mortality rates. Despite the knowledge of previous diseases caused by viruses of the same family, such as MERS and SARS-CoV, management and treatment of patients with COVID-19 is a challenge. One of the best strategies around the world to help combat the COVID-19 has been directed to drug repositioning; however, these drugs are not specific to this new virus. Additionally, the pathophysiology of COVID-19 is highly heterogeneous, and the way of SARS-CoV-2 modulates the different systems in the host remains unidentified, despite recent discoveries. This complex and multifactorial response requires a comprehensive therapeutic approach, enabling the integration and refinement of therapeutic responses of a given single compound that has several action potentials. In this context, natural compounds, such as Curcumin, have shown beneficial effects on the progression of inflammatory diseases due to its numerous action mechanisms: antiviral, anti-inflammatory, anticoagulant, antiplatelet, and cytoprotective. These and many other effects of curcumin make it a promising target in the adjuvant treatment of COVID-19. Hence, the purpose of this review is to specifically point out how curcumin could interfere at different times/points during the infection caused by SARS-CoV-2, providing a substantial contribution of curcumin as a new adjuvant therapy for the treatment of COVID-19.

## Introduction

Coronavirus disease 19 (COVID-19/2019-nCoV) is caused by the severe acute respiratory syndrome coronavirus 2 (SARS-CoV-2). The clinical manifestation of COVID-19 range from asymptomatic upper respiratory tract infection to critical illness and pneumonia associated with acute respiratory distress syndrome (ARDS) (Guan et al., 2020). The main risk factors associated with greater severity and mortality caused by COVID-19 include hypertension, diabetes mellitus, cardiovascular disease (CVD), advanced age, and obesity (Simonnet et al., 2020; Wu and McGoogan, 2020; Zhou et al., 2020).

SARS-CoV-2 is an enveloped β-coronavirus composed of four structural proteins: spike (S), envelope (E), membrane (M), and nucleocapsid (N) proteins (Chen et al., 2020). Entry of the virus into the host cell occurs through the cleavage of protein S into two subunits (S1 and S2) where SARS-CoV-2 develops a multibasic site at the S1-S2 boundary, which is cleaved by furin to form protein S for processing by TMPRSS2 (Hoffmann et al., 2020). The amino-terminal S1 subunit contains a receptor-binding domain (RBD) that is responsible for binding to the cell surface receptor, angiotensin-converting enzyme 2 (ACE2) (Wrapp et al., 2020; Xia et al., 2020). The membrane-anchored S2 subunit is composed of the fusion peptide (FP), heptapeptide repeat sequences 1 and 2 (HR1/HR2), transmembrane domain (TM), and cytoplasmic domain. These components are responsible for viral fusion and cell invasion (Huang Y. et al., 2020; Xia et al., 2020). After the RBD domain is attached to ACE2, the S2 subunit changes its conformation and moves closer to the viral envelope and cell membrane for viral fusion and entry (Huang Y. et al., 2020). In the host, ACE2 is widely expressed in the lungs, heart, liver, vascular endothelium, kidneys, and gut. It is an important regulator of the renin-angiotensin-aldosterone system (RAAS), and promotes the conversion of angiotensin I (Ang I) to Ang (1–9) and Ang II to Ang (1–7) (D’ardes et al., 2020; Gheblawi et al., 2020). Ang (1–7) has an important physiological role and promotes vasodilation, including anti-hypertrophic, anti-inflammatory, anti-oxidant, anti-thrombotic, and anti-fibrotic effects (Imai et al., 2005; Kuba et al., 2005; Chung et al., 2020; D’ardes et al., 2020). The conversion of Ang II to Ang (1–7) regulates the concentration of Ang II-mediated by ACE2. When available, Ang II binds to the ATR1 receptor, thereby promoting harmful pro-inflammatory effects, such as hypertrophy, oxidative stress, and vasoconstriction (Imai et al., 2005; Kuba et al., 2005; Chung et al., 2020; D’ardes et al., 2020). Therefore, the negative regulation of ACE2, promoted by the binding of SARS-CoV-2, results in increased levels of Ang II (Imai et al., 2005; Kuba et al., 2005; D’ardes et al., 2020).

The current drugs approved by the Food and Drug Administration (FDA) for the treatment of patients with COVID-19 prior to the writing of this manuscript are: Fresenius Medical, multiFiltrate PRO System and multiBic/multiPlus Solutions (Fresenius Medical Care); Fresenius Kabi Propoven 2% (Fresenius Kabi USA, LLC.); REGIOCIT replacement solution that contains citrate for regional citrate anticoagulation (RCA) of the extracorporeal circuit (Baxter Healthcare Corporation); COVID-19 convalescent plasma (Office of the Assistant Secretary for Preparedness and Response US Department of Health and Human Services); remdesivir (Veklury) (Gilead Sciences, Inc.); bamlanivimab (Eli Lilly and Company); baricitinib (Olumiant) in combination with remdesivir (Veklury) (Eli Lilly and Company); REGEN-COV (casirivimab and imdevimab) (Regeneron Pharmaceuticals); bamlanivimab and etesevimab (Eli Lilly and Company); and Propofol-Lipuro 1% (B. Braun Melsungen AG), as obtained from the regulators database (https://www.fda.gov/).

Drug repurposing has been viewed as a promising strategy for combating COVID-19. Several factors, such as molecular recognition, binding affinity, and interactions, are calculated during computational drug design and development. Virtual screening was performed with approximately 3,410 drugs approved by the FDA. However, remdesivir was yet to be approved at the time, but has since been analyzed (Beck et al., 2020). The aforementioned and other studies suggested that remdesivir is a potential antiviral agent against SARS-CoV-2, following the demonstration of its affinity to target sites of the virus, including RNA-dependent RNA polymerase (RdRP), helicase, 3-to -5 exonuclease, 2-O-ribose methyltransferase, and endoRNAse from SARS-CoV-2 and SARS-CoV-2 main protease (Mpro, also called 3CLpro) (Beck et al., 2020; Elfiky, 2020). Following this methodology, curcumin displayed promising results, making it a strong candidate for *in vitro* and *in vivo* studies against SARS-CoV-2.

Natural compounds based on medicinal plants and traditional Chinese medicine (TCM) formulas with antiviral action against coronavirus have been investigated. These compounds presented several targets against SARS-Cov and Middle East Respiratory Syndrome (MERS), such as (1) spike (S) glycoprotein, (2) papain-like protease (PLpro), and (3) nucleocapsid (N) proteins. Among these compounds, including the specific viral targets, are ginsenoside-Rb1 (1), hirsutenone (2), tanshinones I–VII (2), with anti-SARS-CoV action, and resveratrol (3) with anti-MERS activity (Wu et al., 2004; Park et al., 2012; Park et al., 2012; Lin et al., 2017). Numerous therapeutic effects of the natural polyphenol, curcumin, have been reported, including potential chemotherapeutic, antioxidant, antiviral, antibacterial, and anti-inflammatory properties (Paciello et al., 2020). Clinical studies have demonstrated the effects of nanoencapsulated curcumin in patients with COVID-19. In the aforementioned study, a significant reduction in clinical manifestations of COVID-19 (fever, cough, and dyspnea) was observed in the group treated with nanocurcumin (patients with mild and severe disease) (Tahmasebi et al., 2020; Valizadeh et al., 2020). In addition, nanocurcumin reduced the mortality rate of these patients. However, the mortality rate of the placebo group was significantly higher than that of the two groups (patients with light and severe disease) treated with nanocurcumin (Tahmasebi et al., 2020; Valizadeh et al., 2020). Currently, another study involving patients with COVID-19 treated with nanoencapsulated curcumin is ongoing (Hassaniazad et al., 2020). Therefore, this manuscript provides a review of the biological effects of curcumin in diseases that arise following SARS-CoV-2 infection.

## *In Silico* Models Predicting the Antiviral Effects of Curcumin Against SARS-CoV-2

**FIGURE 1:**
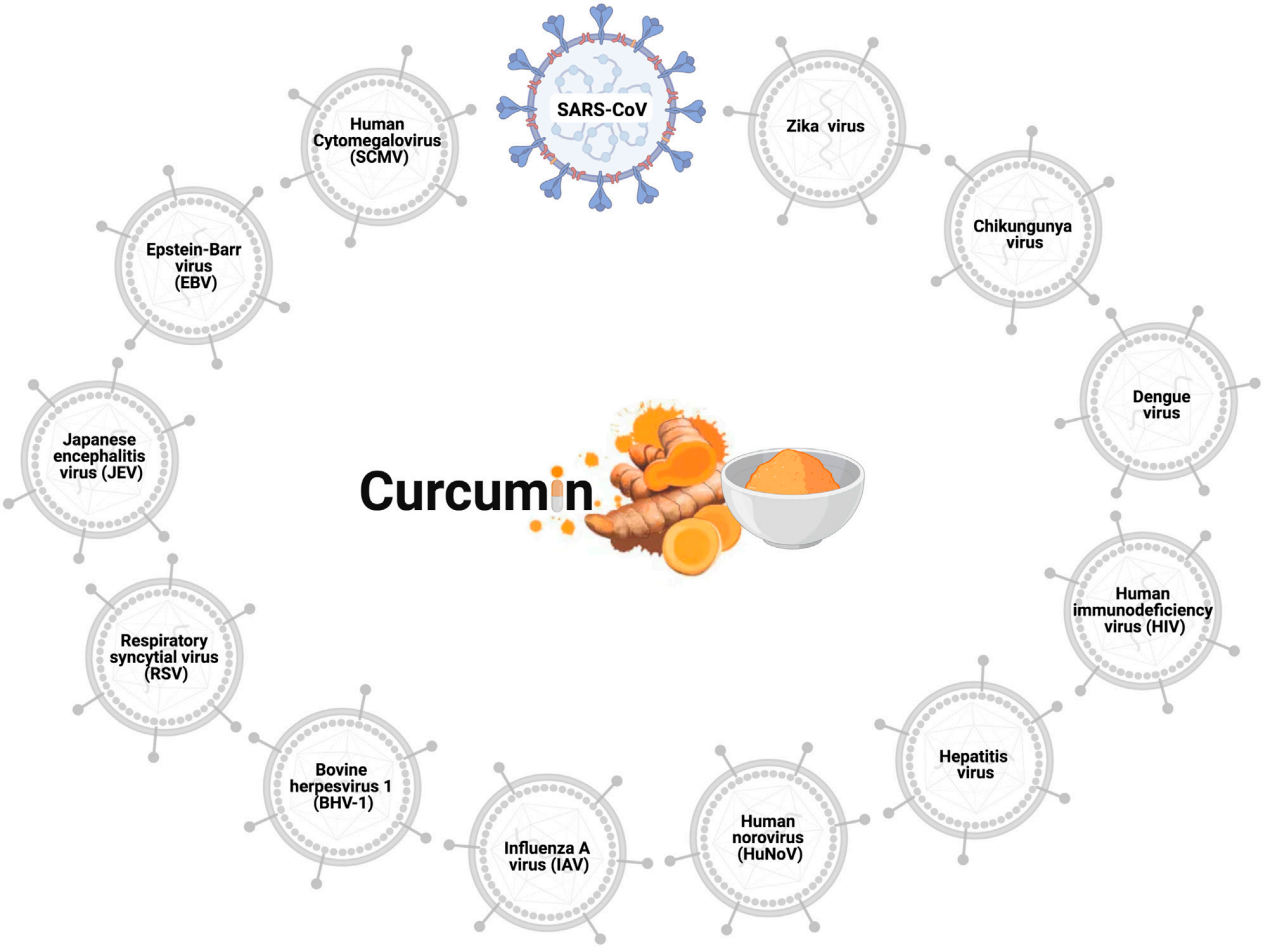
Antiviral effects of curcumin. Curcumin prevents cell infection and viral replication in the SARS-CoV, influenza A virus (IAV), zika virus, chikungunya virus, hepatitis C virus (HCV), human norovirus (HuNoV), viral hemorrhagic septicemia virus in fish (VHSV), bovine herpesvirus 1 (BHV-1), respiratory syncytial virus (RSV), Japanese encephalitis virus (JEV), Epstein-Barr virus (EBV), human cytomegalovirus (HCMV), and human immunodeficiency virus (HIV).

The antiviral effects of curcumin have been widely explored, and the viruses to which curcumin has antiviral action are shown in Figure 1. Curcumin prevents the binding of the influenza A virus (IAV) (Chen et al., 2010; Ou et al., 2013), dengue virus (Balasubramanian et al., 2019), zika virus, and chikungunya virus (Mounce et al., 2017) to host cells. Curcumin inhibits the entry of the hepatitis C virus (HCV) (Chen et al., 2012; Anggakusuma et al., 2014), human norovirus (HuNoV) (Yang et al., 2016), viral hemorrhagic septicemia virus in fish (VHSV) (Jeong et al., 2015), and bovine herpesvirus 1 (BHV-1) (ZHU et al., 2015). Furthermore, the curcumin hinders viral genome replication and transcription of the respiratory syncytial virus (RSV) (Obata et al., 2013; Yang et al., 2016) and Japanese encephalitis virus (JEV) (Dutta et al., 2009), and interferes with the translation and assembly of the Epstein-Barr virus (EBV) (Hergenhahn et al., 2002), human cytomegalovirus (HCMV) (Lv et al., 2014a; Lv et al., 2014b), and human immunodeficiency virus (HIV) (Gupta et al., 2011; Ali and Banerjea, 2016). *In vitro* analyses revealed the antiviral action of curcumin against the SARS-CoV virus in Vero-E6 cells; this natural polyphenol could inhibit viral replication at concentrations of 3–10 µM (Wen et al., 2007). Based on such data regarding antiviral activity, researchers using in silico prediction models evaluated the potential of curcumin against the binding proteins of SARS-CoV-2 and its cellular receptors.

The SARS-CoV-2 S glycoprotein is responsible for the interaction between the virus and the host cell, promoting fusion and internalization of the virus via the ACE2 receptor. Thus, both the S glycoprotein and ACE2 are potential targets for the treatment of COVID-19. *In silico* analysis showed that curcumin has a high-affinity for interaction with the S glycoprotein through the establishment of six hydrogen bonds (Maurya et al., 2020). In this study, curcumin obtained higher scores than the control compounds, such as nafamostat and hydroxychloroquine (Maurya et al., 2020). In addition, curcumin displayed an affinity for ACE2. Moreover, docking results showed that curcumin interacted with the active site of the protein, in addition to forming two hydrogen bonds (Maurya et al., 2020). Similarly, curcumin demonstrated a better affinity for ACE2 than the control compounds, such as captopril and hydroxychloroquine (Maurya et al., 2020).

The transmembrane protein serine protease 2 (TMPRSS2) facilitates the entry of SARS-CoV-2 from the spike protein (Hoffmann et al., 2020). *In silico* analyses focusing on TMPRSS2 showed that curcumin forms four hydrophobic interactions and an H-bond with TMPRSS2 (Motohashi et al., 2020). These findings corroborated results of *in vitro* studies where curcumin treatment led to the downregulation of TMPRSS2 in prostate cancer cells (Zhang et al., 2007; Thangapazham et al., 2008).

The main protease (Mpro) of SARS-CoV-2 is indispensable in maturation and viral replication, and is a promising target in the treatment of SARS-CoV-2. The proteins that are matured by Mpro include RNA-dependent RNA polymerase (RdRp, Nsp12) and helicase (Nsp13), which depend on the cleavage of Mpro (Rut et al., 2020). Inhibition of Mpro prevents viral replication; thus, compounds with inhibitory effects on Mpro have become attractive targets for the treatment of COVID-19 (Zhang S. et al., 2020; Anand et al., 2003). To identify compounds with potential binding to Mpro, an *in-silico* study using docking was carried out to evaluate a series of compounds, including the drugs currently used in the treatment of COVID-19. In this study, two compounds with a high affinity for Mpro were used as controls: N3 and O6K (HUYNH; WANG; LUAN, 2020). Among the compounds tested, including chloroquine, entecavir, hydroxychloroquine, and remdesivir, curcumin surprisingly formed the most stable complex with SARS-CoV-2 Mpro, and the affinity score was comparable to that of the N3 control (Huynh et al., 2020).

The entry of SARS-CoV-2 through the endosome requires an endosomal environment with an acidic pH that is promoted by the endosomal proteases, cathepsin B and L, and ion channels, particularly the vacuolar ATPase pump (V-ATPase), which is crucial in regulating endosomal pH (Aslam and Ladilov, 2020; Khan et al., 2020). Curcumin has been shown to be a potential pH controlling agent, decreasing the expression of V-ATPase, which causes an increase in pH in tumor cells (Vishvakarma et al., 2011).

**FIGURE 2:**
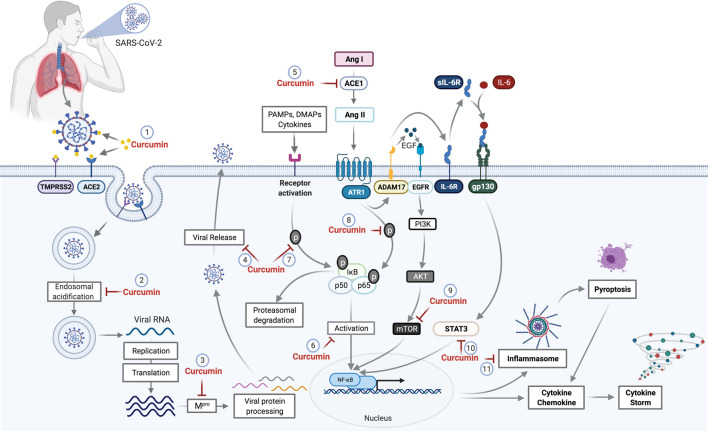
Potential curcumin targets as antiviral and anti-inflammatory in SARS-CoV-2 infection. The first antiviral effect of curcumin against SARS-CoV-2 is its potential for preventing the binding of viral S protein to the ACE2 receptor and initiate the host cell infection process (1). After penetrating the host cell via endosomes, the virus begins the replication process that requires an acid endosomal environment to initiate the proteolytic process of viral proteins and subsequent release to the external environment. Curcumin acts by inhibiting the Endosomal acidification (2) and processing of the viral proteins (Mpro), necessary for viral release (3,4). Further, the inhibition of ACE mediated by curcumin (5) prevents the increase of Ang II levels. Curcumin inhibits NF-κB (6) through the inhibition of different pathways. The binding of PAMPs, DAMPs, and cytokines that leads to IkB phosphorylation and proteasomal degradation is one of those pathways that cause NF-κB activation. Curcumin prevents both IkB phosphorylation and p65 subunit from the NF-κB (8), which consequently prevents NF-κB activation. The activation of ADAM17 by the AngII-ATR1 axis promotes the interaction between EGF and EGFR receptor, which promotes the activation of the PI3K/AKT/mTOR axis resulting in NF-κB activation. Curcumin acts as a potential inhibitor for mTOR (9), preventing the NF-κB pathway activation. ADAM17-mediated signaling also triggers the release of soluble interleukin 6-receptor, forming a complex with IL-6 (sIL-6R-IL-6) that binds to glycoprotein gp130. This complex binding (sIL-6R-IL-6+gp130) activates the signal transduction pathways responsible to induce the activators of transcription 3 (STAT3). Activation of STAT3 results in activation of NF-κB, which can be prevented by the curcumin (10). The NF-κB activation induces a protein complex formation, knowns as inflammasome, which can lead to cell death through pyroptosis, a pathway to cell death mediated by the activation of caspase-1. However, curcumin can cause the inhibition of inflammasome formation (11) by the inhibition of NF-κB. Abbreviations: TMPRSS2, transmembrane protease, serine 2; ACE1, angiotensin-converting enzyme 1; ACE2, angiotensin-converting enzyme 2; Mpro, main protease; PAMPs, pathogen-associated molecular pattern; DAMPs, damage-associated molecular patterns; ANG I, angiotensin I; Ang II, angiotensin II; ATR1, angiotensin II (AII) receptor 1; ADAM17, a disintegrin and metalloproteinase 17; EGF, epidermal growth factor; EGFR, epidermal growth factor receptor; IL-6R, interleukin 6 receptor; sIL-6R, soluble Interleukin 6 receptor; gp130, glycoprotein 130; PI3K, phosphoinositide 3-kinase; AKT, protein kinase B; mTOR, mammalian target of rapamycin; STAT3, signal transducers and activators of transcription; NF-κB, factor nuclear kappa B.

*In vitro* results of the antiviral action of curcumin on SARS-CoV and the data from *in silico* analyses reinforce the hypothesis of the potential activity against SARS-CoV-2. Thus, this review aims to encourage evaluation of the effect of curcumin on cells infected by SARS-CoV-2 and the replication of the virus using *in vitro* and *in vivo* models, and in randomized clinical trials. The possible interaction sites of curcumin with SARS-CoV-2 in the host cells are shown in Figure 2.

## Effects of Curcumin in the COVID-19-Induced Inflammatory Process

The inflammatory process of COVID-19 is complex and multifactorial. Patients with the severe form of the disease can be affected by a hyperinflammatory condition called a cytokine storm, highlighting the need for anti-inflammatory treatment to alleviate the hyperactivation of the immune response, which induces this cytokine storm. Focusing on the anti-inflammatory action of curcumin, two studies were conducted with patients with COVID-19. In the first study, the research group investigated the modulation of pro-inflammatory cytokines by nanocurcumin. Patients with COVID-19 showed high mRNA expression and secretion of cytokines, IL-1β, IL-6, TNF-α, and IL-18, but showed a significant reduction in IL-6 and IL-1β after treatment with nanocurcumin (Valizadeh et al., 2020). Subsequently, exploring the modulatory mechanisms of nanocurcumin, the researchers demonstrated that the number of Th17 cells, gene expression, and serum Th17-mediated factors level (IL-17, IL-21, IL-23, and GM-CSF) were significantly reduced in both stages of the disease in the group of patients with COVID-19 treated with nanocurcumin (Tahmasebi et al., 2020).

Despite the rapid scientific progress regarding the pathophysiology of COVID-19, the precise mechanisms that trigger the exacerbated inflammatory response observed in some of the patients have not yet been completely elucidated. However, several hypotheses attempt to explain such changes. The nuclear factor-kappa B (NF-κB) pathway is directly involved in this inflammatory process and can stimulate the production of pro-inflammatory cytokines when activated. Recent findings led to concerns regarding the overstimulation of the NF-κB pathway and its potential contribution to the emergence of cytokine storms. Studies have shown that NF-κB can be activated directly by SARS-CoV-2 from Toll-like receptors (TLRs) and RAAS system components (Mahmudpour et al., 2020). In such situations, the SARS-CoV envelope (E) and nucleocapsid (N) proteins were shown to be directly related to NF-κB activation (Liao et al., 2005; DeDiego et al., 2014). Consequently, when this protein was deleted in a genetically modified virus, a reduction in NF-κB activation was observed (DeDiego et al., 2014).

Activation of the AngII-AT1R axis causes NF-κB activation (Crowley and Rudemiller, 2017). The AngII-AT1R axis is directly involved in the pro-inflammatory response by acting on the main pathways that lead to the release of cytokines and chemokines. The increase in AngII stimulates the phosphorylation of the NF-κB p65 subunit, leading to its activation and the subsequent release of cytokines (IL-6, IL-1ß, IL-10, and TNF-α) (Ruiz-Ortega et al., 2001; Skurk et al., 2004). The AngII-AT1R axis activates disintegrin and metalloprotease 17 (ADAM17), processing the membrane form of IL-6Rα to its soluble form (sIL-6Rα) through epidermal growth factor (EGFR). The sIL-6Rα-IL-6 complex leads to gp130-mediated STAT3 activation (Eguchi et al., 2018; Murakami et al., 2019), with STAT3 being essential for the complete activation of the NF-κB pathway, in conjunction with the main pathway stimulator, IL-6 (Murakami et al., 2019). The cytokines, TNF and IL-1, also trigger signals that cause the translocation of NF-κB to the nucleus by activating genes involved in the production of inflammatory mediators (Crowley & Rudemiller, 2017). Curcumin blocks STAT3-mediated NF-κB activation, and the consequent reduction in pro-inflammatory cytokines disrupts the positive feedback between pro-inflammatory cytokines and NF-κB (Alexandrow et al., 2012; Rahardjo et al., 2014; Ma et al., 2015; Yadav et al., 2015).

NF-κB is inactive in the cell cytoplasm because of its association with the IκB protein complex. In the presence of stimuli (PAMPs, DAMPs, and cytokines), IκB undergoes phosphorylation and proteasomal degradation that dissociates the NF-κB complex, allowing NF-κB to translocate into the nucleus, leading to the expression of chemokines and pro-inflammatory cytokines (Solt and May, 2008). Curcumin acts by inhibiting the phosphorylation of IκB through inhibiting translocation and the consequent activation of NF-κB (Karunaweera et al., 2015; Wang et al., 2018; Cheemanapalli et al., 2019). Owing to NF-κB inhibition, there is a reduction in the production of inflammatory cytokines, such as IL-1α, IL-6, and TNF-α (Rahardjo et al., 2014; Ma et al., 2015; Yadav et al., 2015).

Viral infections commonly activate inflammasomes. SARS-CoV has been shown to express at least three proteins that activate the NLRP3-type inflammasome (NOD-, LRR-, and pyrin domain-containing protein 3): envelope protein (E), Open Reading Frame-3a (ORF3a), and Open Reading Frame-8b (ORF8b) (Nieto-Torres et al., 2015; Chen et al., 2019; Shi et al., 2019). Protein E and ORF3a stimulate NF-κB signaling, thereby promoting the release of pro-inflammatory cytokines, such as IL-1β, IL-8, and IL-18, and priminf NLRP3 expression to reach the functional level (Kanzawa et al., 2006; DeDiego et al., 2014; Siu et al., 2019). The amino acid sequence of protein E is 94.7% conserved in SARS-CoV and SARS-CoV-2, indicating the possibility of inflammasome activation in patients with COVID-19 (Chan et al., 2020; Lu et al., 2020). A recent study demonstrated that active caspase-1 (Casp1p20), IL-1β, IL-18, IL-6, and lactate dehydrogenase (LDH) were increased in the serum of patients with COVID-19, and that Casp1p20 and IL-18 are products derived from inflammasomes (Rodrigues et al., 2021). The researchers also found active inflammasome NLRP3 in peripheral blood mononuclear cells (PBMCs) and in the tissues of deceased patients at autopsy. The levels of IL-18 and Casp1p20 were higher in patients who had severe disease, indicating a worse prognosis (Rodrigues et al., 2021). Therefore, the regulation of NF-κB by curcumin inhibits the formation of inflammasomes, specifically NLRP3, decreasing the secretion of IL-1β and IL-18 (Yin et al., 2018).

Another regulator of NF-κB is the mammalian target of rapamycin (mTOR) pathway. mTOR is comprised of two complexes, mTORC1, which is sensitive to rapamycin inhibition through the Raptor protein that is associated with mTORC1, and mTORC2, which is associated with Rictor protein, and has low sensitivity to rapamycin (Saxton and Sabatini, 2017). In lipopolysaccharide sepsis models, the inhibition of mTOR by rapamycin resulted in decreased phosphorylation of the p65 subunit of NF-κB, with a consequent reduction in cytokines and pro-inflammatory chemokines, such as IL-1β, IL-18, IL-6, TNF-α, MCP-1, and led to the reduced expression of the NLRP3 inflammasome (Temiz-Resitoglu et al., 2017; Jia et al., 2019). Although rapamycin is already used as an immunosuppressant in the treatment of transplant patients, it has numerous adverse effects and is associated with a high cost. Curcumin is a potential target inhibitor of the mTOR pathway and can promote the inhibition of both the mTORC1 and mTORC2 complexes (Beevers et al., 2009). Curcumin at low doses was found to suppress the mTORC1-Raptor interaction, leading to inhibition of the mTORC1 complex. Curcumin also promoted interruption of the mTORC2-Rictor interaction at higher doses, thereby inhibiting mTORC2 (Beevers et al., 2006; Beevers et al., 2009; Johnson et al., 2009).

The anti-inflammatory mechanisms of curcumin have been extensively investigated in clinical studies of several inflammatory diseases, such as Crohn’s disease, ulcerative proctitis, ulcerative colitis, irritable bowel syndrome, rheumatoid arthritis, postoperative inflammation, gastric ulcer, *Helicobacter pylori* infection, and idiopathic inflammatory orbital pseudotumor (Gupta et al., 2013). Evaluating the mechanisms of action of curcumin already described in both experimental and clinical trials, which can potentially benefit patients with dysregulated immune responses in COVID-19, seems to be an innovative strategy. The mechanisms of action of curcumin and its potential effects on COVID-19 are showed in Figure 2.

## Curcumin in Hemostatic Disorders

A growing number of studies have reported thromboembolic events in patients hospitalized due to COVID-19. High D-dimer levels are considered to be a common marker for increased thrombotic propensity and poor prognosis (Paliogiannis et al., 2020; Zhou et al., 2020). Increased platelet activation and viral RNA detectable in the blood are associated with platelet hyperactivity, leading to abnormal blood clotting. These causes have been associated with thromboembolic prognosis in patients with COVID-19 (Zhang L. et al., 2020). The following signs of hypercoagulability have been observed in these patients: prolonged prothrombin time (PT), activated partial thromboplastin time (APTT), and elevated levels of D-dimer and other fibrin degradation products (FDP) (Tang et al., 2020). In such cases, antithrombin (AT) activity has been reported to be lower than normal (Tang et al., 2020). Human platelets express ACE2 and TMPRSS2 receptors. SARS-CoV-2 binds to these receptors and promotes platelet activation (Zhang L. et al., 2020).

Endothelial cells express the necessary receptors for SARS-CoV-2 to bind and infect cells, causing cell damage and apoptosis. Damage to the vascular endothelium exposes pro-coagulating factors, such as collagen and von Willebrand factor (vWF), and stimulates the release of tissue factor (TF) (Grobler et al., 2020; Iba et al., 2020). Platelets express specific receptors for these molecules, including glycoprotein VI (GPVI) which binds to sub-endothelial collagen, and glycoprotein (GP) Ib-IX-V which binds to vWF (Falati et al., 1999; Grobler et al., 2020). In addition, activated platelets express P-selectin, which binds to monocytes and circulating neutrophils *via* the PSGL-1 receptor, causing activated monocytes to express TF and activated neutrophils (McFadyen et al., 2020). Curcumin exerts a critical antiplatelet effect, preventing platelet adhesion to the vascular endothelium and subendothelium, in addition to reducing the expression of P-selectin and GP VI (Zhang et al., 2008; Mayanglambam et al., 2010).

Activated neutrophils release extracellular neutrophil traps (NETs). This process is accompanied by cell death (NETosis) and can exacerbate the inflammatory response (Schönrich and Raftery, 2016; Bonaventura et al., 2018). NETs can contribute to the formation of clots and thrombi *via* platelet-dependent or independent pathways. The latter can cause total blood vessel occlusion, resulting in organ damage (Jiménez-Alcázar et al., 2017; Gómez-Moreno et al., 2018). Studies have shown that defects in NET degradation cause partial or total obstruction of blood vessels in the lungs (Jiménez-Alcázar et al., 2017). Furthermore, analyses of lung tissue collected at autopsy from patients with acute respiratory distress syndrome and sepsis revealed the presence of NET components in the observed clots (chromatin and myeloperoxidase), indicating that NETs can form intravascular clots in humans (Jiménez-Alcázar et al., 2017). The products released from NETs can also be cytotoxic to endothelial cells, leading to the recruitment of more NETs, which contributes to a thrombo-inflammatory response (Gómez-Moreno et al., 2018). Curcumin treatment, both *in vitro* and *in vivo*, was demonstrated to inhibit the function of NETs and reduce neutrophilic infiltration in a murine air pouch model induced by LPS (Antoine et al., 2013). In addition, the reduction in expression of P-selectin promoted by curcumin may be a key mechanism in the reduction of NETS; this is because platelets use P-selectin to bind to neutrophils, thereby promoting neutrophilic activation (Zhang et al., 2008; McFadyen et al., 2020).

In endothelial cells associated with the airways, the increased concentration of Ang II causes TF to be upregulated, with consequent activation of the pro-coagulant response (Nishimura et al., 1997). TF is expressed after vascular injury or activation of endothelial cells. Inflammatory mediators, such as TNF-α and IL-1β, are important inducers of TF in endothelial cells (Pendurthi et al., 1997). When expressed, TF serves as a receptor for factor VIIa, and the binding of factor VIIa to TF initiates the coagulation cascade. This leads to thrombin generation and sequential clot formation with the deposition of fibrin protofibrils (Hergenhahn et al., 2002; Butenas et al., 2008; D’Alessandro et al., 2018; Sathler, 2020).

Treatment of human endothelial cells with curcumin inhibited the expression of TF induced by TNF-α, LPS, and thrombin (Pendurthi et al., 1997). Curcumin was also found to inhibit platelet aggregation induced by arachidonic acid, adrenaline, and collagen (Srivastava et al., 1995). These findings corroborate those of another study that revealed the inhibition of platelet agonists, viz. epinephrine-induced platelet aggregation, platelet-activating factor (PAF), and arachidonic acid, with curcumin (Shah et al., 1999). Furthermore, curcumin has been shown to inhibit the formation of thromboxane A2 (TXA2) by platelets (Shah et al., 1999). Platelet aggregation is stimulated by TXA2 produced by active platelets, and promotes the activation of other platelets. Pretreatment of platelets with curcumin inhibited platelet aggregation induced by the calcium ionophore A-23187, following curcumin interfering with the mobilization of intracellular Ca^2+^, which is essential for platelet aggregation (Shah et al., 1999). Curcumin has also been shown to decrease the levels of D-dimers, circulating platelets, and inhibit diesel exhaust particles (DEP) (Nemmar et al., 2012).

Curcumin administration in an *in vivo* model of disseminated intravascular coagulation (DIC) reduced the circulating levels of TNF-α, preventing the consumption of peripheral platelets and plasma fibrinogen (Chen et al., 2007). Curcumin also reduced the deposition of fibrin in the renal glomeruli, a characteristic finding of DIC with curcumin (Chen et al., 2007). In a clinical study, a 10 mg curcumin injection administered for 15 days was sufficient to reduce plasma fibrinogen levels (Ramirez Boscá et al., 2000).

**FIGURE 3:**
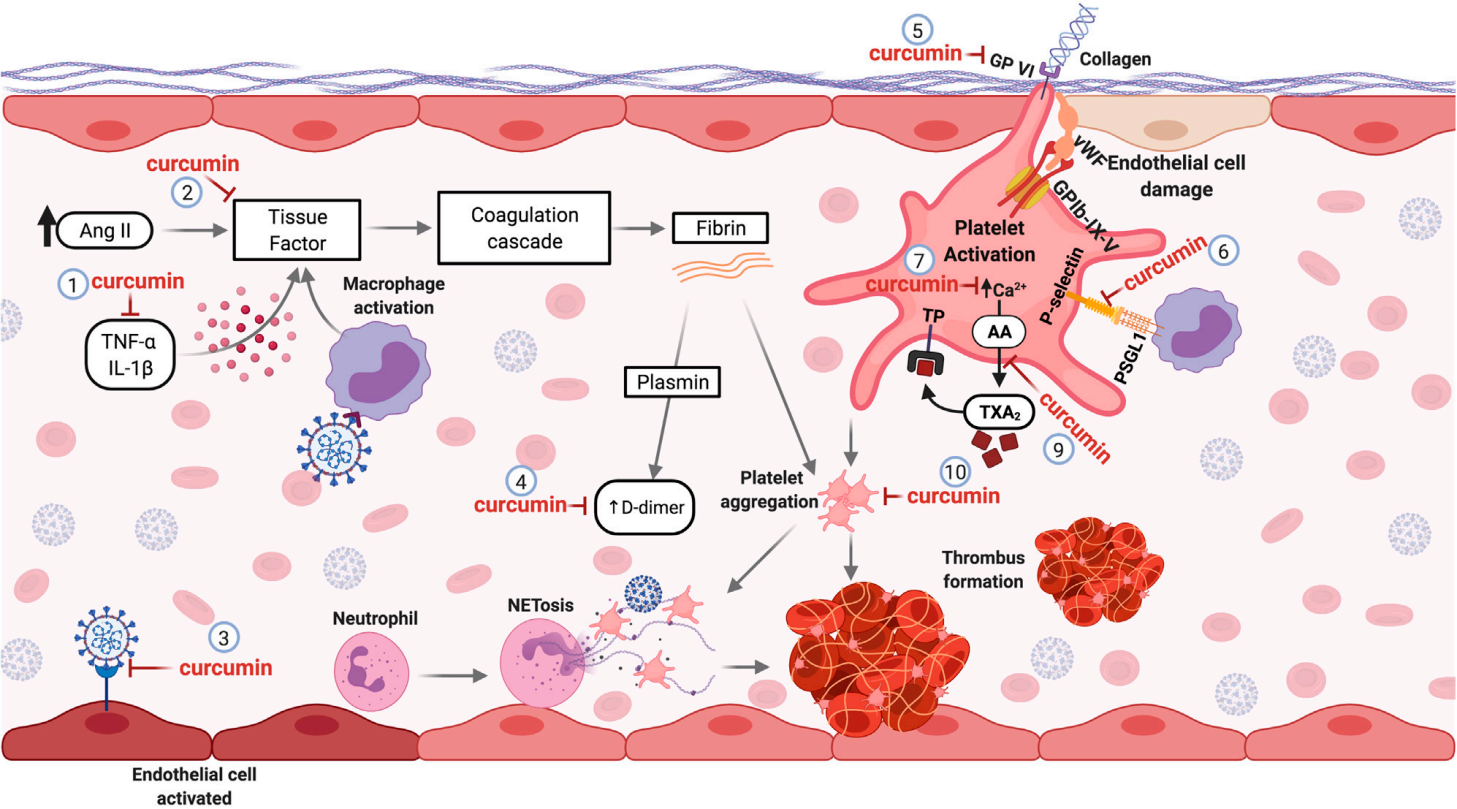
Curcumin as a Potential antithrombotic in hemostatic disorders induced by SARS-CoV-2. Pro-inflammatory cytokines and Ang II elevated levels can induce the production of tissue factor (TF) by the endothelial cells, initiating the coagulation cascade. Curcumin decreases pro-inflammatory cytokines (1) and inhibits TF expression (2) in the vascular endothelium, avoiding the activation of the coagulation cascade. The affinity of curcumin by the SARS-CoV-2 protein S and ACE2 binding can prevent the infection and activation of endothelial cells (3). During the activation of the coagulation cascade, fibrinolysis can occur, generating D-dimers. Curcumin treatment decreases fibrin deposition and D-dimer levels formation (4). Lesions of the endothelial cells can expose the subendothelial collagen, which can be recognized by the platelet’s receptor (GP-VI), leading to platelet cell activation. Curcumin can inhibit the GP-VI receptor, reducing and/or abolishing the platelet activation by binding to collagen (5). The interaction of platelets with monocytes through binding the P-selectin-PSGL-1 receptor promotes monocyte activation, causing an increase of TF expression. Curcumin inhibits this interaction by inhibiting P-selectin in platelets (6). The mobilization of intracellular calcium mediates platelet aggregation. Curcumin prevents calcium-mediated platelet aggregation (7). Besides, curcumin inhibits the thromboxane A2 (TXA2) generation (9) released by activated platelets to stimulate other platelet activation. Thus, curcumin inhibits platelet aggregation (10). Abbreviations: TNF-α, tumor necrosis factor alpha; IL-1β, interleukin 1 beta; Ang II, angiotensin II; GPVI, glycoprotein VI; vWF, Von Willebrand factor; GPIb-IX-V, glycoprotein (GP) Ib-IX-V; PSGL-1, P-selectin glycoprotein ligand-1; AA, arachidonic acid; TXA2, thromboxane A2; TP, thromboxane receptor.

Procoagulant and pro-thrombotic events are recurrent in patients with COVID-19 and can cause significant damage. Curcumin, a well-tolerated natural compound, is a promising candidate for studies in the context of COVID-19 disorder hemostatic. In fact, several *in vitro* and *in vivo* studies have reported its anticoagulant and antithrombotic effects. Therefore, the mechanisms described in the management of other diseases can be reused for new studies regarding hemostatic disorders induced by SARS-CoV-2 deserving further investigation. The molecular mechanisms underlying the targets of curcumin involved thrombotic and coagulant disorders caused by COVID-19 are illustrated in Figure 3.

## Curcumin as a Potential Agent Against Pulmonary Impairment

Alveolar type II (ATII) cells are the primary target of SARS-CoV-2 infection, triggering the apoptotic death of target cells and subsequent infection of adjacent ATII alveolar cells (Mason, 2020). The inflammatory process, together with cellular damage, results in the appearance of multinucleated giant cells and a fibrin-rich hyaline membrane, which causes diffuse alveolar damage that can progress to acute respiratory distress syndrome (ARDS) (Dushianthan et al., 2011). In a model of lung injury induced by benzo (a) pyrene (BaP), curcumin reduced the death of ATII cells and decreased the levels of pro-inflammatory cytokines (TNF-α, IL-6, and C-reactive protein) in serum (Almatroodi et al., 2020).

In more severe cases, patients with COVID-19 may require mechanical ventilation (MV) (Fan et al., 2020). However, inadequate MV can worsen pulmonary pathology. Ventilator-induced lung injury (VILI) causes lung expansion conversion into biochemical signals, resulting in increased activation of inflammatory cells (Silva et al., 2015). Experimentally, it has been shown that curcumin reverses the damage caused by VILI, reducing edema and lung injury. This effect was found to be mediated by the inhibition of NF-κB and the reestablishment of the redox balance from recovery of total antioxidative capacity (Wang et al., 2018).

High levels of circulating NETs have been detected in intubated patients with COVID-19 (Middleton et al., 2020). A correlation between severity and NETs has been established, suggesting that NETs contribute to COVID-19-related lung injury. In addition, platelet colocalization with citrullinated histone H3^+^ and NETs indicated the presence of NETosis in pulmonary microthrombi of patients who died of COVID-19 (Middleton et al., 2020). In the lungs, NETs have a cytotoxic effect on epithelial cells, endothelial cells, and connective tissue, which can aggravate pulmonary pathology (Saffarzadeh et al., 2012). In sepsis and ARDS, NETs cause cell damage and microthrombi, potentially resulting in multiple organ dysfunction and death (Czaikoski et al., 2016; Lefrançais et al., 2018; Papayannopoulos, 2018). In experimental studies involving ARDS due to polymicrobial sepsis (CLP), curcumin decreased the apoptosis of lung cells and attenuated the severity of lung injury. IL-17A acts on ATII cells causing them to release CXCL-1, in turn inducing neutrophil aggregation. Curcumin treatment reduced the levels of IL-17A and neutrophils in the lungs (Chai et al., 2020).

Regulatory T cells (Tregs) are essential regulators of the inflammatory process and generate an adequate immune microenvironment through their anti-inflammatory and anti-apoptotic functions (Lin et al., 2018). Curcumin induces the differentiation of naïve CD4^+^ T cells to Tregs by regulating the expression of IL-10 (Chai et al., 2020). IL-10 is an anti-inflammatory cytokine that promotes macrophage reprogramming from an inflammatory profile (M1) to a repeating profile (M2) by suppressing the mTORC1 complex. M2 macrophages decrease the inflammatory process and stimulate tissue repair in sepsis-induced LPA (Ip et al., 2017). Macrophages with the M1 phenotype are essential for controlling viral replication. However, limiting immunopathological reactions through the M2 phenotype is essential (Sang et al., 2015). In a COVID-19 study, severely ill patients showed a higher frequency of type M1 macrophages than patients with moderate infection or healthy control subjects who presented higher frequencies of type M2 macrophages (Liao et al., 2020). Curcumin promotes a decrease in M1 and an increase in M2 macrophages in septic lungs, indicating its potential effect on macrophage polarization (Chai et al., 2020).

In an *in vivo* model of lung injury mediated by cyclophosphamide, treatment with curcumin reduced lung injury and restored the oxidant-antioxidant balance by reducing lipid peroxidation (Ashry et al., 2013). In LPS-induced acute lung injury (ALI), treatment with curcumin decreased pulmonary edema, increased PaO_2_, and improved lung function (Cheng et al., 2018). ALI can be a consequence of hemorrhagic shock and resuscitation (HSR). Animals subjected to HSR and treated with curcumin showed a reduction in the levels of reactive oxygen species, TNF-α, and neutrophilic infiltrates. Such finding indicates that the treatment provided a protective pulmonary barrier function (Yu-Wung Yeh and Wang, 2020). ALI and ARDS studies in animals with sepsis showed that treatment with curcumin attenuated lung damage and decreased proinflammatory cytokine levels (Xiao et al., 2012; Xu et al., 2013; Liu et al., 2017).

**FIGURE 4:**
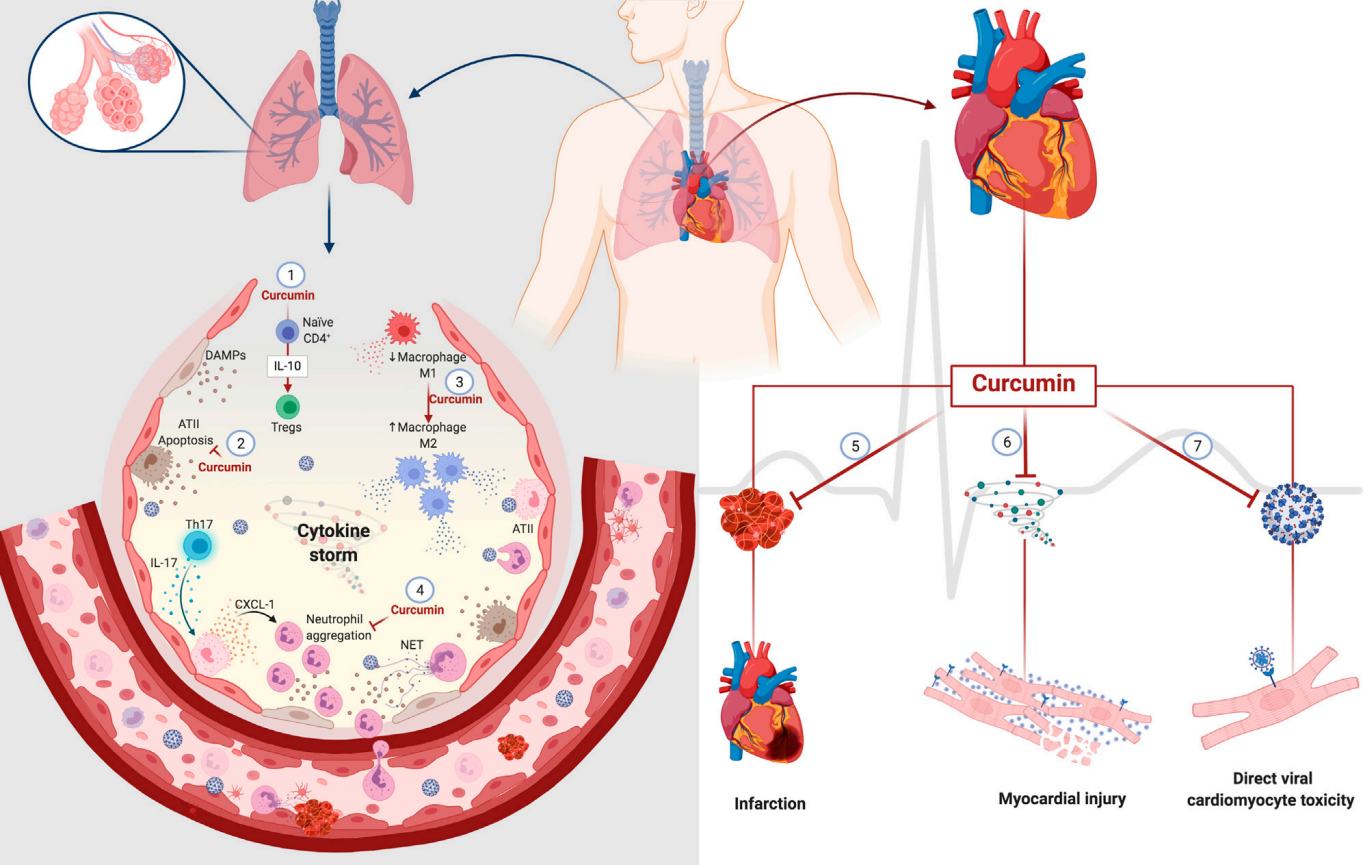
Potential curcumin in cell damage caused by SARS-CoV-2 in the lung and heart. Curcumin promotes differentiation from naïve CD4+T-cell to Tregs through the modulation of IL-10 (1). The cytoprotective role of curcumin decreases the death of type II alveolar cells (ATII) with a consequent decrease in the release of DAMPs (2). Curcumin also mediates macrophages' polarization, decreasing the population of inflammatory macrophages M1 to macrophages M2 that participate in the resolving and reparative process (3). The presence of Th17 cells promotes the activation of ATII cells through IL-17. In turn, activated ATII cells release a chemoattractant for neutrophils that causes neutrophil aggregation. Curcumin decreases IL-17 levels with a consequent decrease in neutrophil aggregates. The anticoagulant and antithrombotic effects of curcumin can have protective effects on the heart, decreasing the heart attack risk (5). The anti-inflammatory action of curcumin can prevent damage to cardiomyocytes caused by an excess of inflammatory mediators, known as a cytokine storm (6). Its affinity for protein S and ACE2 can prevent the direct infection of cardiomyocytes by SARS-CoV-2 (7). Abbreviations: ATII, alveolar type II cells; Tregs, regulatory T cells; Th17, T helper 17 cells; CXCL-1, chemokine ligand 1; NET, neutrophil extracellular traps.

Although clinical studies have not reported the direct effects of curcumin on respiratory impairment, the decrease in clinical manifestations (fever, cough, and dyspnea) in patients with COVID-19 is a promising indicator that encourages further investigations (Tahmasebi et al., 2020; Valizadeh et al., 2020). Many clinical trials have established the therapeutic potential of curcumin, either as a single agent or in combination with other drugs in various diseases, owing to its effect on diverse cell signaling pathways. The possible curcumin action sites that can be targeted after SARS-CoV-2-induced changes in the lungs are illustrated in Figure 4.

## Cardioprotective Effects of Curcumin

Clinical reports involving some of the first patients with COVID-19 from the Wuhan province of China showed that 5 of the 41 patients had changes in levels of highly sensitive cardiac troponin I (hs-cTnI), indicating myocardial injury (Huang C. et al., 2020). Interestingly, some patients sought medical assistance after cardiac symptoms (palpitations and chest tightness) rather than the classic symptoms of COVID-19 (fever and cough) (Deng et al., 2020; Stefanini et al., 2020). In children, COVID-19 can cause a hyperinflammatory syndrome similar to Kawasaki disease (Riphagen et al., 2020).

Underlying CVD significantly increases the mortality rate of patients with COVID-19. One study showed that patients with COVID-19, CVD, and increased troponin T levels had a mortality rate of 69.4%; however, the mortality rate of patients with COVID-19 with increased levels of troponin T without CVD was 37.5% (Guo et al., 2020).

The cardiac events reportedly caused by COVID-19 include acute myocardial injury, heart failure, acute coronary syndrome, infarction, and arrhythmia (Lang et al., 2020; Amirfakhryan and Safari, 2021). The hypotheses surrounding cardiovascular involvement in COVID-19 involve direct infection of cardiac cells by SARS-CoV-2, injury mediated by the inflammatory process, reduced oxygen supply, hypoxia, microthrombi, and stress cardiomyopathy (Lang et al., 2020; Amirfakhryan and Safari, 2021). Histopathological analysis of the heart of a patient with COVID-19 revealed cardiac tissue with a fibrin thrombus in a perforating vein associated with myocardial infarction, myocardial necrosis (transmural), and neutrophilic infiltrates (Rapkiewicz et al., 2020).

In experimental models of sepsis, curcumin proved to be effective at improving the survival parameters, reducing hypovolemia levels observed in the late phase of sepsis, suppression of hyperglycemia in the acute phase, and attenuation of hypoglycemia in the late stage (Silva et al., 2017). Curcumin also attenuated heart damage induced by sepsis; improved cardiac function and body temperature (Yang et al., 2013); and reduced troponin I levels and the product of lipid peroxidation, suggesting its reduction of oxidative damage (Yang et al., 2013).

The restoration of blood flow in the ischemic myocardium can exacerbate tissue injury and result in a poorly adaptive tissue process (Vinten-Johansen et al., 2005; Prasad et al., 2009). First, oxidative stress activates metalloproteinases (MMPs) that promote degradation of the extracellular matrix (ECM). This results in the progressive expansion of the infarction, thinning of the ventricular wall, and dilation of the chamber (Wang et al., 2012). The cure for the infarction involves deposition of collagen, forming a fibrotic and non-functional scar. In an experimental model of ischemia and reperfusion, treatment with curcumin reduced ECM degradation by MMPs and increased the synthesis of collagen and the accumulation of myofibroblasts (Wang et al., 2012). Consequently, there was an improvement in cardiac function, reduced left ventricle dilation, and increased wall thickness (Wang et al., 2012).

An increased number of studies evaluating post-COVID-19 sequelae warns of cardiovascular symptoms, such as chest pain and palpitations (Schneider, 2020; Carvalho-Schneider et al., 2021; Halpin et al., 2021; Huang et al., 2021; Vallejo et al., 2021). The cumulative incidence of thrombosis (2.5% at 30 days after discharge), including segmental pulmonary embolism, intracardiac thrombus, thrombosed arteriovenous fistula, and ischemic stroke, were reported in a single-center study in the United States with 163 patients (Patell et al., 2020). The 6-month post-evaluation of COVID-19 showed that patients suffer from long-term sequelae of the disease, including venous thromboembolic diseases (cardiovascular and cerebrovascular events) (Huang et al., 2021). Currently, there are no reports of curcumin in cardiac changes resulted from COVID-19. However, based on data published on other diseases and cardiac disorders, we hypothesize that curcumin may be a promising agent in preventing cardiovascular damage caused by SARS-CoV-2 infection, as summarized in Figure 4.

## Conclusion

Due to the uncountable mechanisms of action addressed in this and other reviews, it has been reinforced that curcumin could serve as an adjuvant drug in COVID-19 treatment (Babaei et al., 2020; Manoharan et al., 2020; Roy et al., 2020; Soni et al., 2020; Zahedipour et al., 2020; Saeedi-Boroujeni et al., 2021; Thimmulappa et al., 2021). The multiplicity of pathophysiological responses induced by SARS-CoV-2 highlights the need for a combination of different drugs as a treatment strategy (i.e., there is no single "magic pill" for the cure of COVID-19). Curcumin is a well-tolerated natural compound in humans, even at high concentrations (Dhillon et al., 2008; Kanai et al., 2011; Gupta et al., 2013). Thus, its combination with drugs that are already approved for use appears logical. Curcumin is a well-tolerated natural compound in humans, even at high concentrations (Dhillon et al., 2008; Kanai et al., 2011; Gupta et al., 2013). Thus, its combination with drugs that are already approved for use appears logical. The first results from the studies regarding the effect of curcumin in patients with COVID-19 are promising. However, several questions need to be answered: 1) Does curcumin prevent SARS-CoV-2 infection of the host cells? 2) Does curcumin treatment attenuate respiratory and cardiovascular system commitment? 3) Is the curcumin able to reestablish hemostatic homeostasis?

Despite the absence of specific studies addressing the mechanism of action of curcumin in the treatment of COVID-19, currently, the world is experiencing an uncommon situation, which has led researchers and physicians to evaluate the available knowledge to the other diseases, in an attempt to design more promising pathways against SARS-CoV-2. In conclusion, this review strategically contributes to the relentless search for therapies that can act on combat of COVID-19, in addition to providing targets for future studies using the curcumin as an adjuvant treatment to COVID-19.
